# Research on Elastic and Elastic-Plastic Buckling Load of Cylindrical Shell with an Inclined through Crack under Axial Compressive Load

**DOI:** 10.3390/ma16186123

**Published:** 2023-09-08

**Authors:** Zhuo-Wu Wang, Jian Tang, Shou-Chao Li, Xiao-Hua He, Chang-Yu Zhou

**Affiliations:** 1 School of Mechanical and Power Engineering, Nanjing Tech University, Nanjing 211816, China; 2 Jiangsu Key Lab of Design and Manufacture of Extreme Pressure Equipment, Nanjing 211816, China

**Keywords:** axial load, buckling experiment, finite element method, inclined through-crack, buckling failure

## Abstract

By experimental methods, 26 specimens were designed to conduct elastic and elastic–plastic buckling tests on cylindrical shells containing cracks. This study discusses the influence of factors such as the length–diameter ratio, the diameter–thickness ratio, the crack length, the inclination of the crack, etc., on the buckling load. Additionally, finite element models were established to compare with experimental results. For the PMMA cylindrical shell, the results showed that as the length–diameter ratio of the cylindrical shell increased, the buckling load first decreased and then increased. For the 6063 aluminum alloy cylindrical shell, with increasing length–diameter ratio, diameter–thickness ratio, and crack length of the cylindrical shell, the buckling load decreased accordingly. However, concerning the crack inclination, as the crack inclination increased, the buckling load increased accordingly. This indicates that the larger the crack inclination, the higher the load capacity of the cylindrical shell containing cracks. Through finite element simulations of cylindrical shells with cracks, it was found that through compressive mechanical properties, both elastic and elastic–plastic buckling loads yielded results that are closer to the experimental results. Additionally, the inclusion of contact effects in numerical simulations further improved the agreement with the experimental results, and the variation trend of the buckling load in the finite element simulation was consistent with the experimental results. The research findings provide valuable references for the assessment of load capacity in structures containing cracks.

## 1. Introduction

The thin-walled cylindrical shell, as a fundamental component, has been widely used in engineering fields such as civil engineering, aerospace, chemical, and shipbuilding [1]. Typically, different brittle and ductile materials are selected according to manufacturing processes and service conditions. Ductile materials are used to manufacture pressure vessels, including tower equipment and submarine pipelines, while brittle materials are employed for pressure vessels like dryers and cooling tower shells [2,3]. Thin-walled cylindrical shells usually need longer service life. Under cyclic loads or mechanical loads, cracks in the shell continuously propagate, leading to a decrease in structural load capacity and even structural failure and serious safety accidents [4].

During the production and service of components, the generation of cracks is inevitable. In engineering, crack sizes and orientations are random, which determines that the cracks belong to mixed-mode cracks [5]. Cracks affect the strength of structures significantly. Equipment subjected to external pressure under extreme conditions has a high level of potential danger. Under the influence of structure size, shell thickness, material properties, and geometric defects, elastic or elastic–plastic buckling is likely to occur [6,7]. In order to accurately and reasonably assess the reliability of structures with cracks, research on the load capacity of structures with crack defects is conducted. Understanding the failure of structures with cracks is of great scientific significance and engineering application value.

Kia Badamchi [8] chose cold-rolled steel ST12 as the material to manufacture with a welding line for axial compression and external pressure buckling tests. They considered the influence of different diameter–thickness ratios on the elastic–plastic buckling load. Geometric defects resulting from the manufacturing process were scanned using displacement sensors and incorporated into the finite element model for elastic–plastic buckling analysis. The results were highly consistent with the experimental results under external pressure and axial compression. Chao Zhang [9] studied two types of cylindrical shells, one made of aluminum and the other made of CFPR, using both experimental and numerical simulation methods based on Hashim’s failure criteria, a cohesive damage model, and non-linear buckling theory. They extensively investigated the compression performance and failure modes of the aluminum cylindrical shell and optimized the CFPR cylindrical shell model. The results showed that the experimental values for the aluminum cylindrical shell were only 15% lower than the theoretical values. Shariati [10] used an INSTRON8802 servo-hydraulic machine to conduct buckling tests under combined loads on low-carbon steel cylindrical shells and considered the impacts of cracks with various shell lengths, diameters, crack lengths, relative positions, and crack inclinations on the results. They also performed elastic–plastic buckling analysis using ABAQUS software and validated the accuracy of the experimental results. Saemi [11] performed elastic–plastic buckling analysis on cylindrical shells containing cracks using ABAQUS software. They discussed the influence of shell length, crack length, relative position of the crack, and crack inclination on the buckling load of the cylindrical shell. They used low-alloy steel as the experimental material and validated the results with numerical simulations, showing good consistency. Huakun Wang [12] investigated the buckling performance of 316L stainless steel seamless tubes with pitting corrosion. Their study revealed that the surface translation of the cylindrical shell and the shape and size of the pitting defect are important parameters that influence the buckling load of the cylindrical shell. Huiyun Qiao [13] considered the effect of the number of cracks based on the elastic–plastic buckling theory and fracture mechanics and designed cylindrical shells made of Q235A steel with one, two, and three end cracks. They used finite element simulation to validate the accuracy of the designs and evaluated the effects of major factors such as the length of the long crack, the length of the short crack, the number of cracks, and the crack spacing on the results. They also introduced adjustment coefficients to estimate the load capacity. Starnes [14] conducted axial compression buckling tests on thin-walled cylindrical shells made of 2024-T3 aluminum alloy with longitudinally oriented long cracks. They also developed a finite element model to study the relationship between axial compression buckling and crack propagation. The results indicated that the buckling load of the shell under axial compression decreased with an increase in the initial crack length. For relatively short initial cracks, the initial buckling led to overall instability or collapse of the shell. In contrast, for longer initial cracks, the initial buckling was a stable localized response mode. Rahman Seif [15] focused on numerical simulation to study the influence of factors such as crack size, cylindrical panel dimensions, and boundary conditions on the buckling load under tension and compression loads. The results showed that the crack size of the cylindrical panel had significant effects on the buckling load. Peng Feng [16] conducted axial compression and bending tests on carbon fiber composite cylindrical shells and plotted force–lateral displacement curves. Subsequently, they simulated the model using finite element software, and the simulation results showed high agreement with the experimental results. Finally, they adopted the Perry–Robertson formula as the fundamental design form to determine the curves. Vaziri [17] conducted linear eigenvalue simulations on cylindrical shells containing through-thickness cracks based on linear elastic buckling theory. They extensively explored the influence of factors such as crack size, crack orientation, and other parameters on the compressive capacity of cylindrical shells under combined axial compression and internal pressure. They found that local buckling in the cylindrical shell typically occurs before overall buckling and internal pressure contributes to the stability of the cylindrical shell.

In the past few decades, research on the buckling problem of cylindrical shells has mainly focused on cylindrical shells or those with geometric defects such as dents and pittings. Although there have been research achievements in the buckling behavior of cylindrical shells containing cracks under axial compressive loads, the influence of brittle materials on cylindrical shells with cracks has not received sufficient attention. Various influencing factors, such as wall thickness and boundary conditions, on the axial compressive buckling failure behavior of ductile materials with cracks in cylindrical shells, have not been fully explored.

This paper focuses on cylindrical shells containing inclined cracks and adopts an experimental approach to investigate the influence of factors such as the length–diameter ratio, diameter–thickness ratio, crack length, and crack inclination on the elastic and elastic–plastic buckling loads. Furthermore, the experimental specimens are numerically simulated using the finite element software ABAQUS 2021 by C3D8R eight-node hexahedral element, and a comparative analysis is performed between the experimental and finite element simulation results of the buckling failure. This paper conducted a study on elastic and elastic–plastic buckling failure from the following aspects:

(1) Conducting material mechanical properties tests to provide mechanical properties parameters for numerical simulations.
(2) Studying the influence of the length–diameter ratio and crack inclination on the elastic buckling load of cylindrical shells containing inclined cracks.
(3) Studying the influence of the length–diameter ratio, the diameter–thickness ratio, the crack length, and the crack inclination on the elastic–plastic buckling load of cylindrical shells containing inclined cracks.

## 2. Test Methods and Schemes of Cylindrical Shells with Inclined Cracks

### 2.1. Test Equipment

**Figure 1:**
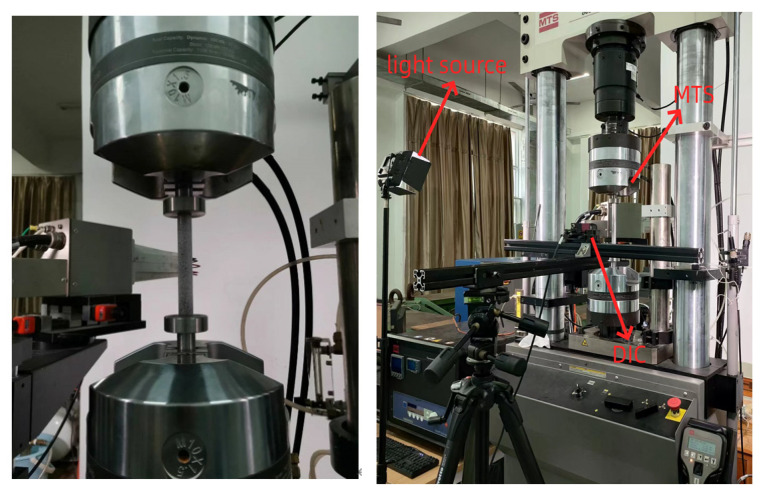
Testing system.

The MTS-809 testing machine (Eden Prairie, MN, USA) used in the experiments is shown in Figure 1, with a maximum normal load of 100 KN and a stroke of 200 mm. A full-field strain measurement system based on the digital image correlation (DIC) method from the Belgian company MATCHID (Gent, Belgium) was used to measure the deformation field of the specimens.

### 2.2. Test Specimens

**Figure 2:**
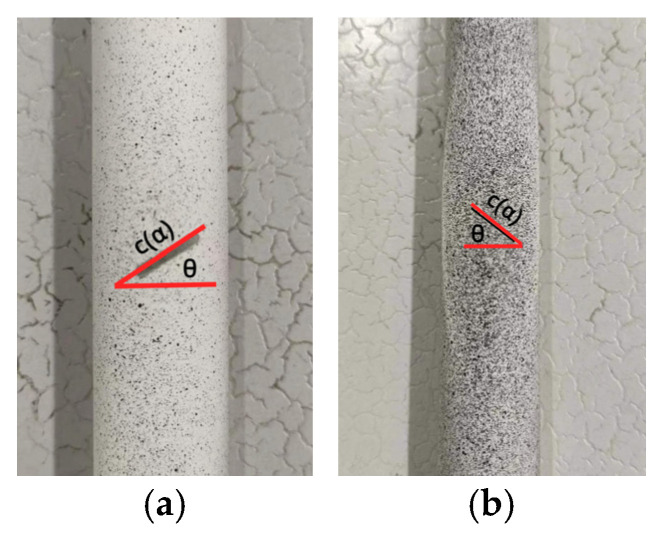
Test specimens for buckling load (α = 60°, θ = 45°): (**a**) PMMA cylindrical shell specimen; (**b**) 6063 aluminum alloy cylindrical shell specimen.

In order to obtain the buckling load of cylindrical shells, this study conducted experiments using cylindrical shell specimens made from two different materials. The elastic buckling test was performed using PMMA cylindrical shells (China), while the elastic–plastic buckling test was conducted using 6063 aluminum alloy cylindrical shells (China). The specimens are shown in Figure 2.

**Figure 3:**
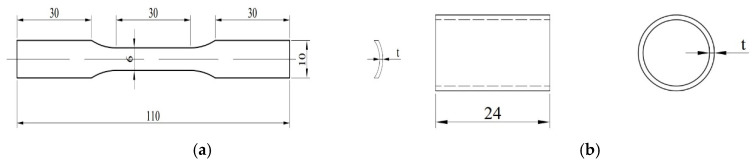
Diagram of tensile and compressive specimens with different thicknesses (t = 1.25, 1, 0.75, 0.5) (unit: mm): (**a**) tensile; (**b**) compress.

In order to ensure the accuracy of subsequent finite element simulation, tensile and compressive mechanical property tests on PMMA and 6063 aluminum alloy were conducted, which could provide the material performance data required for finite element calculations. The standard dimensions of the tensile specimens are determined according to ASTM E8 “Standard Test Methods for Tension Testing of Metallic Materials” [18], shown in Figure 3a. The compression specimens are designed according to “GBT7314-2017 Room Temperature Compression Test Methods for Metallic Materials” [19], shown in Figure 3b.

### 2.3. Testing Program

#### 2.3.1. Experimental Scheme for Elastic Buckling Failure of Cylindrical Shells with Cracks under Axial Compressive Load

Cylindrical shell specimens are made from PMMA material, as shown in Table 1. The effects of cylindrical shell length and crack angle on elastic buckling load were considered. In order to reduce the error caused by measurement in the test, two specimens of each size were made for the experiments, and the average value was taken as the result.

#### 2.3.2. Experimental Scheme for Elastic–Plastic Buckling of Cylindrical Shells with Cracks under Axial Compressive Load

The cylindrical shell specimens are made from 6063 aluminum alloy, shown in Table 2. The effects of length–diameter ratio, diameter–thickness ratio, crack length, and crack angle on elastic–plastic buckling load of cylindrical shell specimens were investigated. The result is treated in the same way as the elastic buckling experiment.

## 3. Determination of Mechanical Property Parameters of Materials

The mechanical properties of materials obtained from tensile and compressive tests usually exhibit significant differences that cannot be ignored. In such cases, if the tensile mechanical performance parameters are used to simulate and calculate the mechanical behavior under compressive load, it may result in deviations.

**Figure 4:**
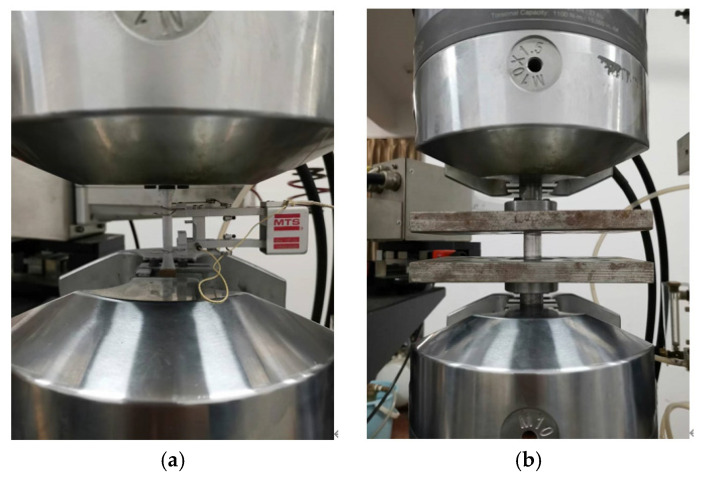
Measurement of mechanical properties of materials: (**a**) tensile test; (**b**) compression test.

Tension and compression tests of specimens were performed with the MTS-809 testing machine. The tension test system and compression test system are shown in Figure 4.

The test results are shown in Table 3 and Table 4. The tensile elastic modulus of 6063 aluminum alloy is 72.44% higher than its compressive elastic modulus, while the compressive elastic modulus of PMMA is 17.89% higher than its tensile elastic modulus. The compressive yield strength of 6063 aluminum alloy is 37.25% higher than its tensile yield strength, while the compressive yield strength of PMMA is 50.4% higher than its tensile yield strength. The compressive ultimate strength of 6063 aluminum alloy is 40.64% higher than its tensile ultimate strength, while the compressive ultimate strength of PMMA is 53.16% higher than its tensile ultimate strength. To investigate the influence of tensile and compressive elastic modulus and yield strength on buckling load, this study employed finite element simulation to calculate the buckling load of cylindrical shells under both tensile and compressive mechanical properties.

## 4. Elastic Buckling Failure Tests of Cylindrical Shells with Cracks

**Figure 5:**
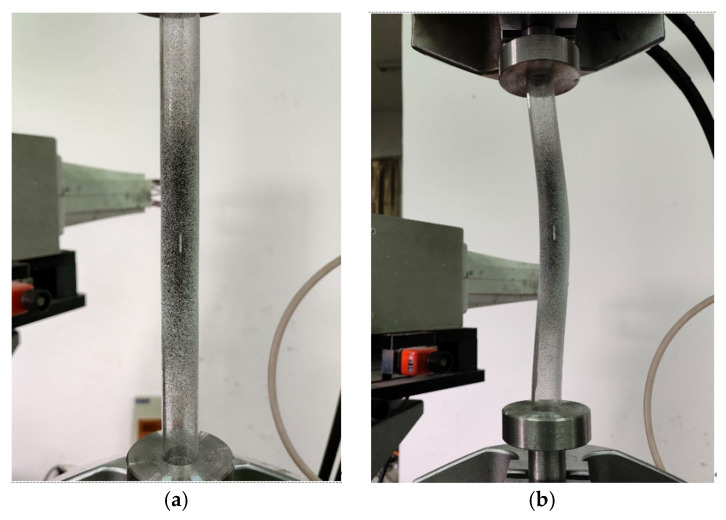
Specimen (B-3) buckling test: (**a**) before buckling test; (**b**) during buckling test.

The PMMA specimen parameters are shown in Table 1. The boundary conditions are set as simply supported at both ends (allowing axial rotational freedom). The testing machine’s loading speed is set at 0.0005 mm/s. The compressive load is slowly applied to the PMMA specimen. The shape of the specimen before and after buckling during the testing process is illustrated in Figure 5.

**Figure 6:**
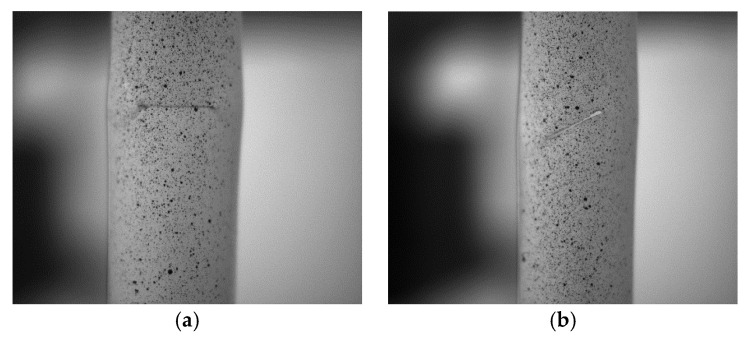
Specimen (A-1, A-2) buckling test: (**a**) specimen (A-1); (**b**) specimen (A-2).

Due to the limit in processing conditions, the crack gap is close to 1 mm wide for the PMMA cylindrical shell. Finite element simulations were conducted to calculate the elastic buckling load of the PMMA cylindrical shell, considering the crack morphology. The experimental results (Exp) were compared with the finite element simulation results, as shown in Figure 6. The finite element simulations took into account the influence of elastic modulus and boundary conditions. In the A Group of experiments, as depicted in Figure 6, specimens A-1 and A-2 exhibited significant crack closure during the compression process. Therefore, in the finite element simulations for specimens A-1 and A-2, the effect of crack surface contact was considered. In the simulations, FEM (E-cs) utilized the elastic modulus obtained from compression tests, while FEM (E-ts) used the elastic modulus obtained from tensile tests. The boundary conditions were set as simply supported at both ends, allowing axial rotational freedom.

**Figure 7:**
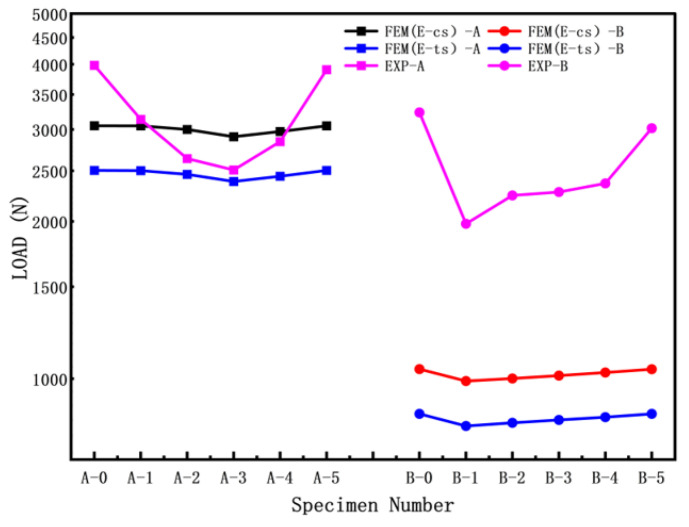
Buckling loads of cracked cylindrical shells.

From Figure 7, it can be observed that the buckling load test values and calculation values of the cylindrical shells without cracks (A-0, B-0) are both higher than those of the cylindrical shells with cracks. In the Group A specimens (shorter cylindrical shells), when the crack inclination angle ≤ 45°, due to the influence of crack closure and crack surface contact, the buckling load of FEM (E-cs) and FEM (E-ts) initially decreases and then increases with an increase in the crack inclination angle. In contrast, the buckling load of Group B (longer cylindrical shells) specimens shows a monotonic increase. For the specimens in Group B, the buckling load of FEM (EP-cs) is much higher than that of FEM (EP-ts). The buckling loads considering compressive properties by finite element simulation are clearly higher than those considering tensile properties. Generally, variation ranges of buckling load by experiment are much greater.

**Figure 8:**
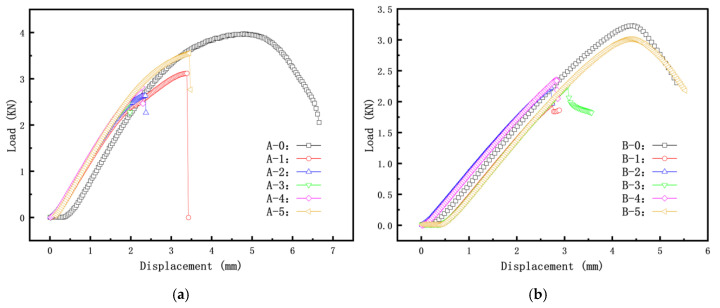
Load–displacement curves for specimens Group A and Group B: (**a**) Group A specimen; (**b**) Group B specimen.

Figure 8 presents the load–displacement curves for different crack inclination angles of cylindrical shells with cracks (cylindrical shells with two lengths). It can be observed that as the length–diameter ratio of the cylindrical shell increases, the load–displacement curve gradually decreases, and the buckling load decreases accordingly. For the shorter cylindrical shells (Group A), the load–displacement curves initially decrease and then increase with an increase in the crack inclination angle. In contrast, for the longer cylindrical shells (Group B), the load–displacement curves gradually increase with an increase in the crack inclination angle. The trends in buckling load–displacement by experiment are consistent with the results obtained from finite element simulations.

The results indicate that specimens A-1 and A-2 showed significant crack surface contact, which increased the experimental buckling load. As a result, the shorter cylindrical shells (Group A) exhibited a trend of initially decreasing and then increasing. On the other hand, the buckling load of longer cylindrical shells (Group B) consistently increased monotonically. The simulation results based on compression performance were found to be closer to the experimental buckling load results.

## 5. Elastic–Plastic Buckling Failure Test of Cylindrical Shells with Cracks

**Figure 9:**
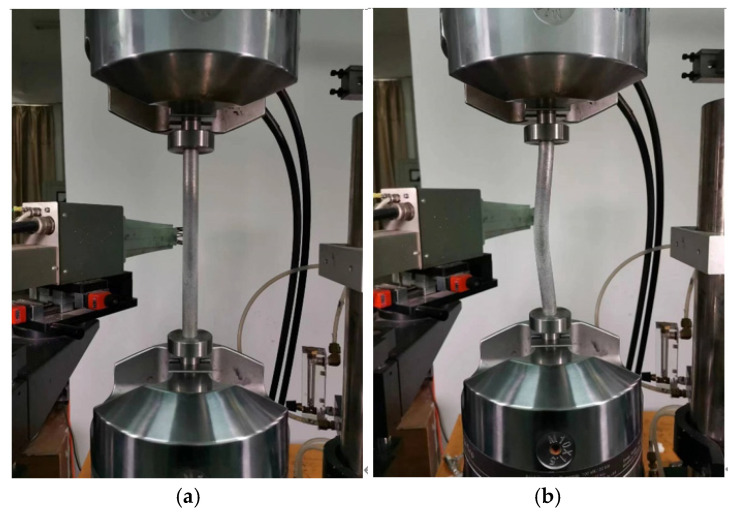
Specimen (H-0) buckling test: (**a**) before buckling test; (**b**) during buckling test.

The mechanical parameters of the 6063 aluminum alloy specimen are shown in Table 2. The boundary conditions and loading speed are the same as before, and the buckling deformation of the specimen is shown in Figure 9.

### 5.1. Influence of Length–Diameter Ratio on Elastic–Plastic Buckling Load

**Figure 10:**
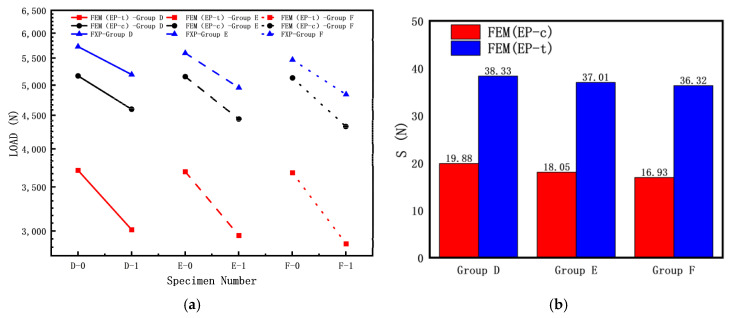
Influence of length–diameter ratio of cracked cylindrical shell on buckling loads: (**a**) FEM and experiment results; (**b**) discrete error between FEM and experiment results.

To investigate the influence of the length–diameter ratio on the elastic–plastic buckling load of cylindrical shells with cracks, the following specimen dimensions were used: R = 8 mm; T = 0.5 mm; crack length c (α = 60°); crack inclination angle θ = 45°; and L/R ratios of 15, 20, and 25. The experimental results and finite element simulation results are shown in Figure 10a, while the discrete errors between the finite element simulation results and experimental results are shown in Figure 10b.

The introduction of the discrete error S describes the degree of agreement between finite element simulation results and experimental data, reflecting the influence of the elastic modulus in the experiment. It is defined as follows:

$$S=\sqrt{\frac{\|{FEM-Exp\|}_{2}}{n}}$$

In the equation, *FEM* represents a set of finite element simulation values, *Exp* represents a set of experimental values, and *n* represents the number of samples in a data set.

From Figure 10, it can be observed that the buckling load test values of the three specimens without cracks (D-0, E-0, F-0) are significantly higher than those of the specimens with cracks. As shown in Figure 10a, the experimental buckling load is higher than the calculated buckling load because the calculation did not consider crack surface contact. During the axial compression test, the crack surfaces of the cylindrical shells with cracks gradually close, resulting in a higher experimental buckling load compared to the calculated buckling load. For the three sets of specimens, the calculated buckling load using FEM (EP-c) is higher than the calculated buckling load using FEM (EP-t). As indicated in Figure 10b, for the Group D specimens, the calculated buckling load using FEM (EP-c) is closer to the experimental results than the calculated buckling load using FEM (EP-t), suggesting that using the compressive mechanical properties for calculating the elastic–plastic buckling load yields results closer to the experimental results.

**Figure 11:**
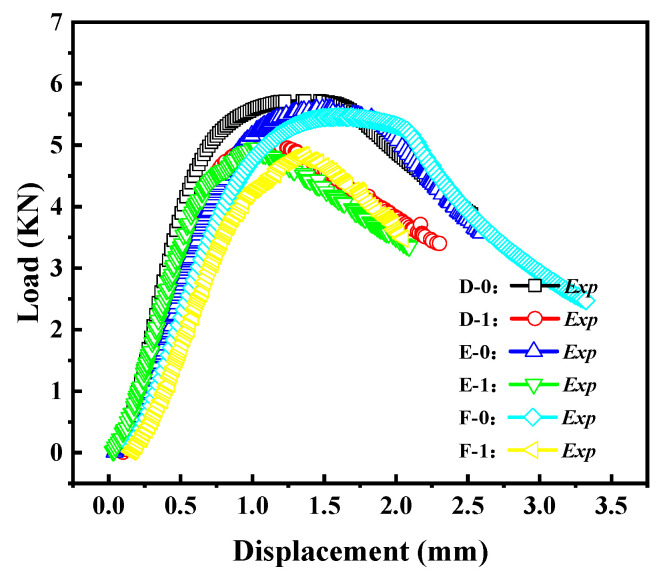
Load–displacement curves of cracked cylindrical shells with different length–diameter ratios.

Figure 11 reflects the load–displacement curves of cylindrical shells with cracks for different shell lengths. It can be observed that as the length–diameter ratio of the cylindrical shell increases, the load–displacement curve gradually decreases, and the buckling load decreases accordingly. This indicates that shorter cylindrical shells have higher load capacity.

### 5.2. Influence of the Ratio of Diameter to Thickness on Elastic–Plastic Buckling Load

**Figure 12:**
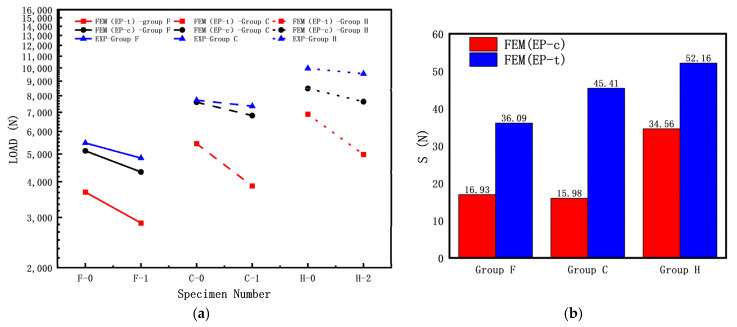
Influence of diameter–thickness ratio of cracked cylindrical shell on buckling loads: (**a**) FEM and experiment results; (**b**) discrete error between FEM and experiment results.

To investigate the effect of the diameter–thickness ratio on the elastic–plastic buckling load of cylindrical shells with cracks, the following specimen dimensions were used: L = 200 mm; R = 8 mm; crack length c (α = 60°); crack inclination angle θ = 45°; and R/T = 8, 10.7, 16. The experimental results and finite element simulation results of the buckling load are shown in Figure 12a, while the discrete errors between the finite element simulation results and experimental results of the buckling load are shown in Figure 12b.

From Figure 12, it can be observed that the buckling load test values of the three specimens without cracks (F-0, C-0, H-0) are significantly higher than those of the specimens with cracks. As shown in Figure 12a, the experimental buckling load is higher than the calculated buckling load, and for all three groups of specimens, the calculated buckling load using FEM (EP-c) is higher than the calculated buckling load using FEM (EP-t). Based on the results shown in Figure 12b, for the Group D specimens, the calculated buckling load using FEM (EP-c) is closer to the experimental results than the calculated buckling load using FEM (EP-t), indicating that the elastic–plastic buckling load based on the compressive mechanical properties are closer to the experimental results.

**Figure 13:**
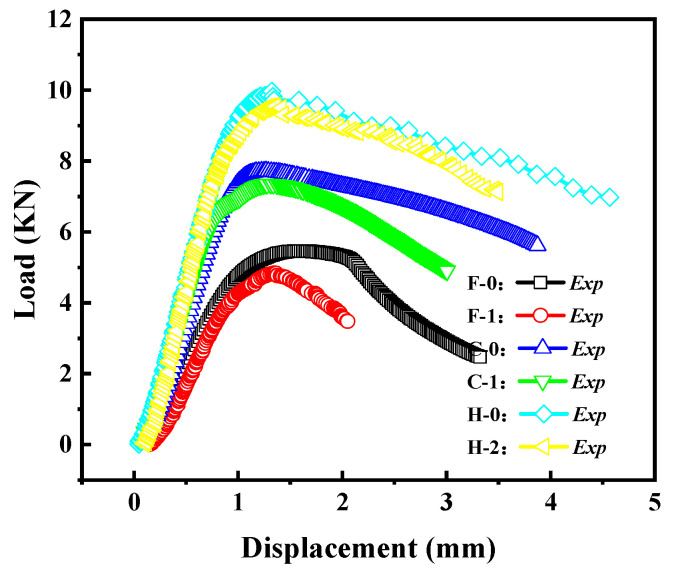
Load–displacement curves of cracked cylindrical shells with different diameter–thickness ratios.

Figure 13 reflects the load-displacement curves of cylindrical shells with cracks at different thicknesses (Group F, C, H). It can be observed that as the diameter–thickness ratio of the cylindrical shell increases, the load–displacement curve gradually decreases, and the buckling load significantly decreases. This indicates that as the thickness of the cylindrical shell increases, its load capacity also increases.

### 5.3. Effect of Crack Length on Elastic–Plastic Buckling Load

**Figure 14:**
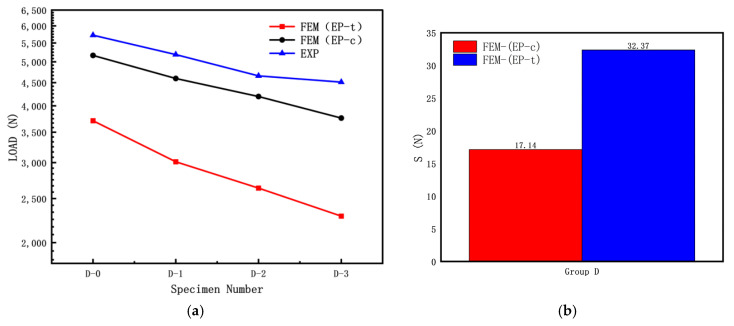
Influence of crack length of cracked cylindrical shell on buckling loads: (**a**) FEM and experiment results; (**b**) discrete error between FEM and experiment results.

To investigate the effect of crack size on the elastic–plastic buckling load of cylindrical shells with cracks, the following specimen dimensions were used: L = 120 mm, R = 8 mm, T = 0.5 mm, crack inclination angle θ = 45°, and crack lengths c (α = 60°, α = 90°, α = 120°). The experimental results and finite element simulation results of the buckling load are shown in Figure 14a, while the discrete errors between the finite element simulation results and experimental results of the buckling load are shown in Figure 14b.

From Figure 14, it can be observed that in the Group D specimens, the buckling load test value of the specimen without cracks (D-0) is significantly higher than that of the specimens with cracks. As shown in Figure 14a, the experimental buckling load is higher than the calculated buckling load, and for the Group D specimens, the calculated buckling load using FEM (EP-c) is higher than the calculated buckling load using FEM (EP-t). Based on the results shown in Figure 14b, for the Group D specimens, the calculated buckling load using FEM (EP-c) is closer to the experimental results than the calculated buckling load using FEM (EP-t), indicating that using the compressive mechanical properties for calculating the elastic–plastic buckling load yields results closer to the experimental results.

**Figure 15:**
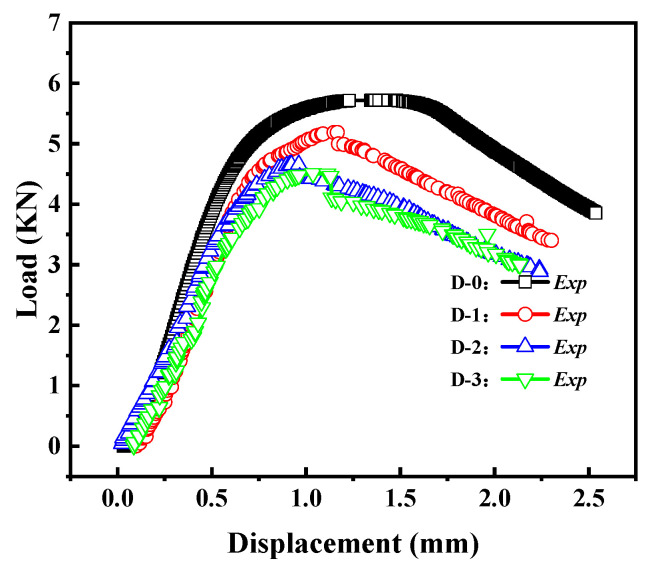
Load–displacement curves of cracked cylindrical shells with different crack lengths.

The load–displacement curves of the Group D specimens are shown in Figure 15. From the graph, it can be observed that as the crack length increases, the load–displacement curve gradually decreases, and the buckling load decreases accordingly. Additionally, with the increase in crack length, the post-buckling stage of the load–displacement curve also gradually decreases.

### 5.4. Effect of Crack Angle on Elastic–Plastic Buckling Load

**Figure 16:**
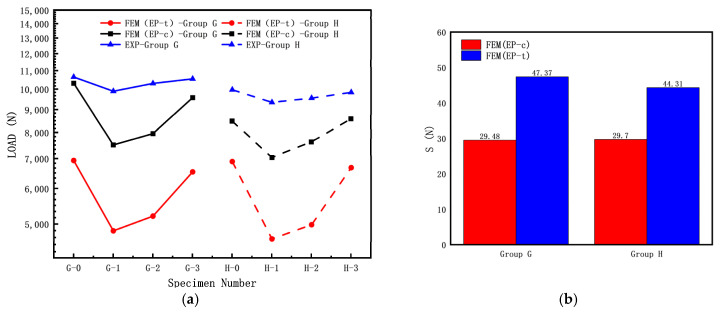
Influence of crack angle of cracked cylindrical shell on buckling loads: (**a**) FEM and experiment results; (**b**) discrete error between FEM and experiment results.

To investigate the effect of crack inclination angle on the elastic–plastic buckling load of cylindrical shells with cracks, the following specimen dimensions were used: L = 120 mm and 200 mm; R = 8 mm; T = 1 mm; crack length c (α = 60°); and crack inclination angle θ = 0°, 45°, 90°. The experimental results and finite element simulation results of the buckling load are shown in Figure 16a, while the errors between the finite element simulation results and experimental results of the buckling load are shown in Figure 16b.

From Figure 16, it can be observed that in the Group G (L = 120 mm, shorter cylindrical shell) and Group H (L = 200 mm, longer cylindrical shell) specimens, the buckling load test values of the specimens without cracks (G-0, H-0) are significantly higher than those of the specimens with cracks. Both the Group G and H specimens show that the calculated buckling load by FEM (EP-c) is higher than the calculated buckling load by FEM (EP-t). Based on the results shown in Figure 16b, for both the Group G and H specimens, the calculated buckling load by FEM (EP-c) is closer to the experimental results than the calculated buckling load by FEM (EP-t); the elastic–plastic buckling load also indicates that the calculated loads by compressive mechanical properties are closer to the experimental data (results). The variety range of elastic and elastic–plastic buckling loads under the same parameters are some different, which is similar to the results in reference to Li [20]. Variational trends in the elastic–plastic buckling load for Group G are consistent, which is different from that elastic buckling load for Group A. In contrast, the trends in Group H and Group B are similar.

**Figure 17:**
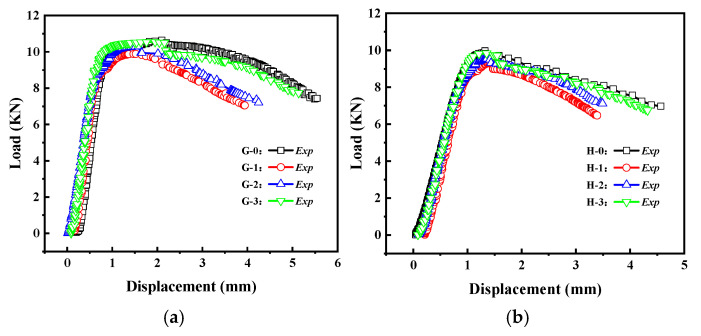
Load–displacement curves of cracked cylindrical shells with different crack angles: (**a**) Group G specimens; (**b**) Group H specimens.

The load–displacement curves of the Group G and H specimens are shown in Figure 17. From the graph, it can be observed that as the crack inclination angle increases, the load–displacement curve gradually rises, and the buckling load increases accordingly. This indicates that a larger crack inclination angle results in a higher load capacity for cylindrical shells with cracks. The crack inclination angle also has a certain influence on the post-buckling stage of the load–displacement curve.

### 5.5. Comparison of Buckling Modes

With the development of computer and image processing technology, the digital image correlation (DIC) technique has emerged due to its advantages of convenient equipment operation, high spatial resolution, and the ability to measure strains in a non-contact manner [21,22]. In order to obtain the deformation of the cracked specimen, a non-contact strain measurement technique was employed in this study to measure the deformation and the strain field of the cylindrical shell with cracks.

**Figure 18:**
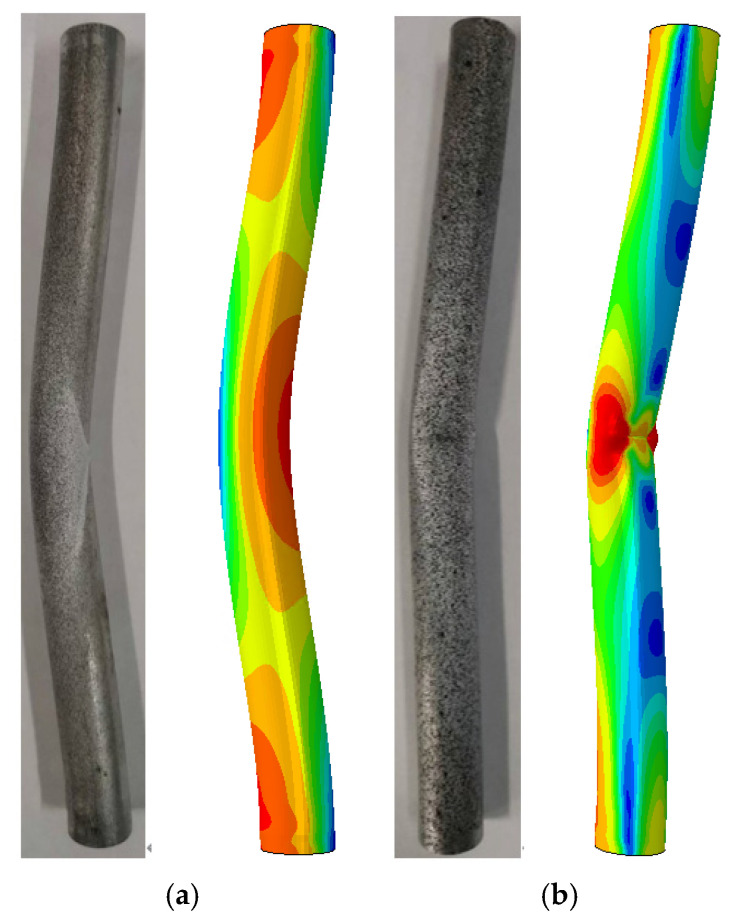

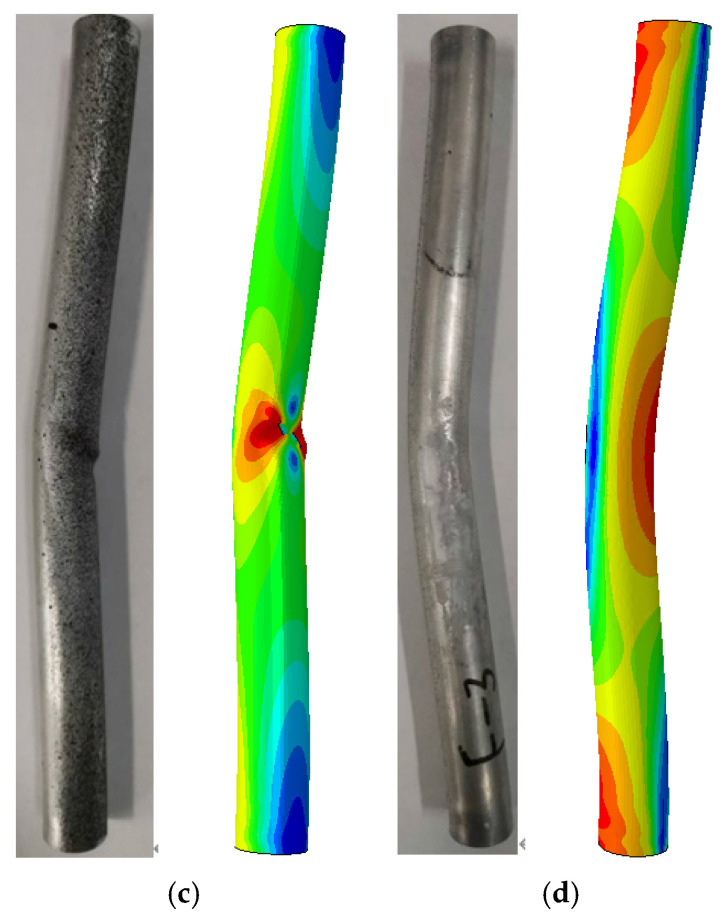
Comparison of buckling modes for Group H by experiment and FEM results: (**a**) H-0 specimen; (**b**) H-1 specimen; (**c**) H-2 specimen; (**d**) H-3 specimen.

In this section, the buckling mode shapes of the cylindrical shell with cracks under different crack inclination angles from Group H were selected and compared with the buckling mode shapes obtained from finite element analysis, as shown in Figure 18. It can be observed that in the axial compression test, the cracks at different inclination angles all experience contact, and the experimental buckling mode shapes are consistent with the calculated buckling mode shapes, indicating the reliability of the experiments.

## 6. Conclusions

This paper investigates the elastic buckling failure and elastic–plastic buckling failure of cylindrical shells with cracks under axial compressive loading through experiments and compares the experimental results with finite element analysis. The main conclusions are as follows:

(1) The tensile and compressive mechanical performance tests were conducted for two materials, PMMA and 6063 aluminum alloy. This study revealed significant differences in the mechanical properties between tension and compression. The finite element result calculated by compressive properties is closer to the experiments.
(2) Under axial compressive loading, the elastic buckling tests of PMMA containing a crack revealed that buckling load by the compressive elastic modulus are closer to the experimental buckling loads. When the length–diameter ratio is small (shorter cylindrical shell) and the crack inclination angle is ≤45°, the crack closes and makes contact, resulting in a trend of decreasing and then increasing buckling load with increasing crack inclination angle. On the other hand, when the length–diameter ratio is large (longer cylindrical shell), the buckling load increases with increasing crack inclination angle, which overall agrees with the results of the elastic buckling load calculations.
(3) Under axial compressive loading, the elastic–plastic buckling tests of 6063 aluminum alloy containing a crack showed that elastic–plastic buckling load by the compressive mechanical properties are closer to the experimental results. The experimental buckling load varies with the length–diameter ratio, the diameter–thickness ratio, the crack length, and the crack inclination angle, and these variations are consistent with the results of the elastic–plastic buckling load calculations.
(4) Considering the effect of buckling loads by length-diameter ratio for the shorter cylindrical shell, the elastic buckling load is greatly affected by boundary conditions, which is different from the elastic–plastic buckling load; however, the trend in the elastic buckling load is consistent with the elastic–plastic buckling load for the longer cylindrical shell.

## Figures and Tables

**Table 1:** Specimens for elastic buckling test.

Specimen Material	Specimen Number	Length of Cylindrical Shell L (mm)	Diameter of Cylindrical Shell R (mm)	Thickness t (mm)	Crack Length c (α) (°)	Crack Angle θ (°)	Exp (N)
PMMA	A-0	120	8	1.25	0	0	3980.3
	A-1				60	0°	3137.75
	A-2				60	30°	2639.09
	A-3				60	45°	2509.71
	A-4				60	60°	2844.04
	A-5				60	90°	3906
	B-0	200			0	0	3234.6
	B-1				60	0°	1980.0
	B-2				60	30°	2243.39
	B-3				60	45°	2277.2
	B-4				60	60°	2366.16
	B-5				60	90°	3017.0

**Table 2:** Specimens for elastic–plastic buckling test.

Specimen Material	Specimen Number	Length of Cylindrical Shell L (mm)	Diameter of Cylindrical Shell R (mm)	Thickness t (mm)	Crack Length c (α) (°)	Crack Angle θ (°)	Exp (N)
6063 Aluminum alloy	C-0	200	8	0.75	0	0	7716.5
	C-1				60	45°	7350.2
	D-0	120		0.5	0	0	5723.6
	D-1				60	45°	5188.4
	D-2				90	45°	4657.1
	D-3				120	45°	4511.3
	E-0	160			0	0	5594.0
	E-1				60	45°	4956.6
	F-0	200			0	0	5464.4
	F-1				60	45°	4839.4
	G-0	120		1	0	0	10,640.2
	G-1				60	0°	9890.0
	G-2				60	45°	10,295.9
	G-3				60	90°	10,538.9
	H-0	200			0	0	9967.4
	H-1				60	0°	9341.9
	H-2				60	45°	9548.0
	H-3				60	90°	9834.5

**Table 3:** Mechanical properties obtained by tensile tests.

Material	Specimen Number	Mean Value (E/*σ_s_*/*σ_b_*)
Aluminum alloy (6063)	t = 0.5	44,766.08/141.11/151.63
	t = 0.75	36,164.62/127.83/158.29
	t = 1	34,474.95/128.29/151.17
PMMA	t = 1.25–4	2051.67/32.48/37.96

**Table 4:** Mechanical properties obtained by compressive tests.

Material	Specimen Number	Mean Value (E/*σ_s_*/*σ_b_*)
Aluminum alloy (6063)	t = 0.5	12,336.01/200.63/225.03
	t = 0.75	15,941.73/203.71/261.39
	t = 1	13,992.48/180/254.66
PMMA	t = 1.25	2498.74/65.48/81.05

## Data Availability

The data that support the findings of this study are available from the corresponding author upon reasonable request.
